# T1 signal intensity ratio variability based on sampling strategies in the pancreas of children and young adults

**DOI:** 10.1007/s00261-024-04774-y

**Published:** 2025-01-27

**Authors:** Arjun K. Mathur, Jonathan R. Dillman, Maisam Abu-El-Haija, David S. Vitale, Jean A. Tkach, Andrew T. Trout

**Affiliations:** 1 University of Cincinnati College of Medicine, Cincinnati, USA; 2 Cincinnati Children’s Hospital Medical Center, Cincinnati, USA

**Keywords:** Children, Pancreatitis, T1-weighted, Signal intensity ratio, MRI, Region of interest

## Abstract

**Purpose:**

T1-weighted signal intensity ratios (SIR) comparing pancreas to spleen (SIRps) or muscle (SIRpm) can semiquantitatively assess T1 signal change associated with pancreatitis. However, there is no standardized methodology for generating these ratios. We set out to determine the impact of MRI sequence as well as region of interest (ROI) location, shape, and size on T1 SIR.

**Methods:**

Retrospective analysis of T1-weighted MR images from 118 patients acquired 2018–2023. A single observer placed ovoid ROIs in the pancreas body/tail and head/uncinate, spleen, and left erector spinae muscle and large irregular ROIs in the pancreas tail and spleen. ROIs were placed on images from two sequences: 3D radial 2 point mDIXON RF spoiled gradient recalled echo sequence (radial) and breath-hold 3D 2-point mDIXON RF spoiled gradient echo (BH). T1 SIR were calculated from mean signal intensity, and agreement was calculated with intraclass correlations coefficients (ICC) and Bland–Altman difference analyses.

**Results:**

118 participants, 57 (48%) female, with mean age 13.7 ± 5.6 years (48%) were included. Agreement was good for SIRps based on irregular versus round ROIs (radial: ICC = 0.90; BH: ICC = 0.91). Agreement was moderate for SIR based on sampling the pancreas body/tail versus head/uncinate (ICC = 0.67–0.76) and poor to moderate based on reference organ (muscle vs. spleen) (ICC = 0.41–0.61). Between sequences, agreement was moderate (ICC = 0.55–0.72, mean difference 0.04–0.09).

**Conclusion:**

The size and shape of the ROI used to sample the pancreas does not meaningfully change T1 SIR but the location sampled, the reference organ used, and the MRI sequence used meaningfully change T1 SIR, potentially impacting disease diagnosis and staging.

**Graphical abstract:**

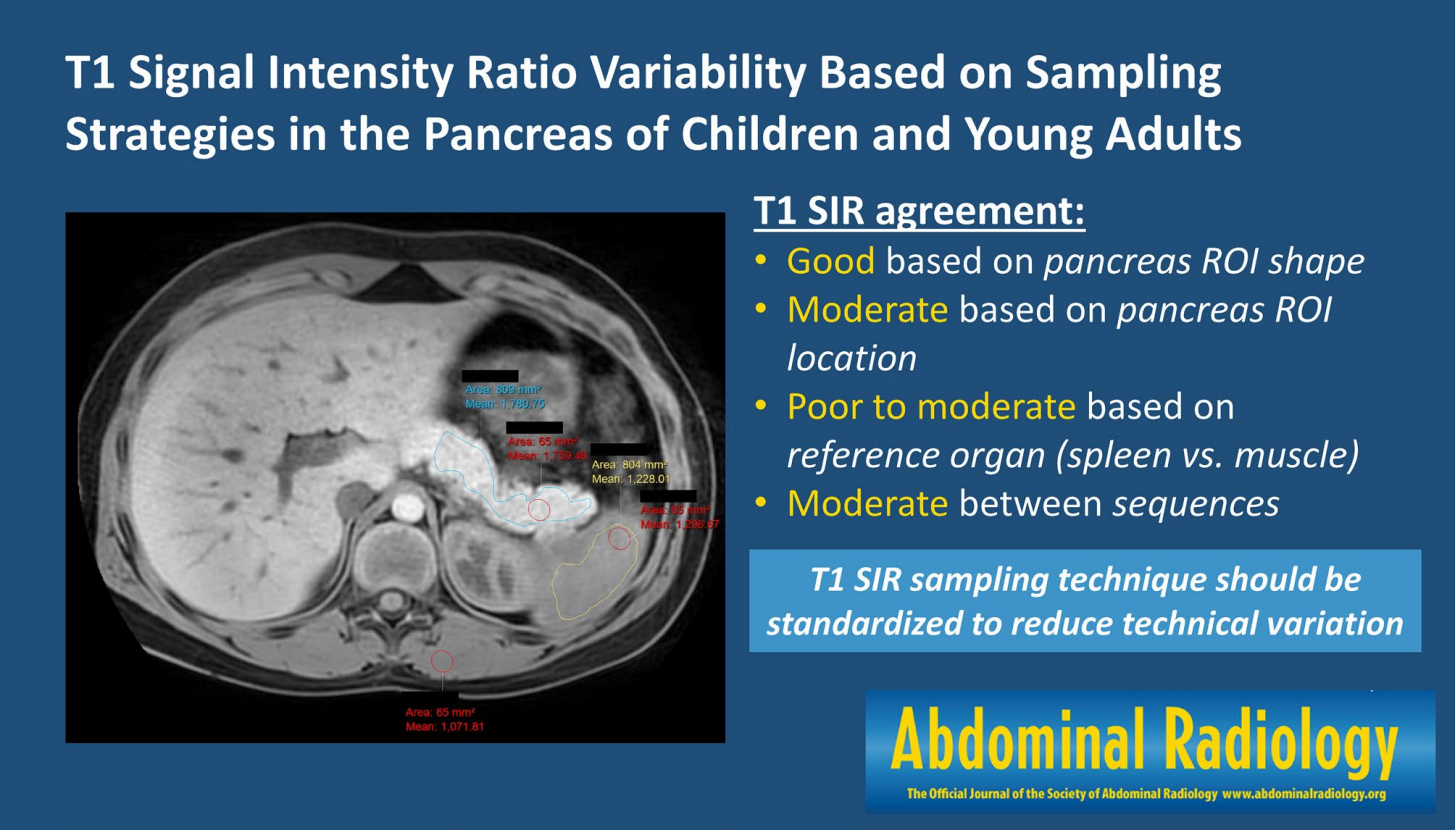

## Introduction

In the past decade, the incidence of pediatric pancreatitis has been increasing such that it now overlaps with the lower end of the incidence in adults [1]. The accepted diagnostic criteria for chronic pancreatitis in children is a combination of imaging findings and clinical findings such as abdominal pain or evidence of exocrine or endocrine pancreatic insufficiency [2]. Imaging features of chronic pancreatitis have not been uniquely defined for children and instead have been extrapolated from findings described in adults. Many of the extrapolated findings are subjective and limited by interobserver variability [3]. Because of this subjectivity, there is an interest in quantitative or semiquantitative findings that can diagnose pancreatitis with a high degree of reproducibility.

Signal intensity ratios (SIR) from T1-weighted MR images have been explored as one potential biomarker and diagnostic tool for pancreatitis in both children and adults [4, 5]. T1 SIR are a semiquantitative measure of T1 signal intensity based on comparison to a reference organ (e.g. spleen or muscle) [6]. This allows assessment of relative T1 signal without performing true T1 relaxation mapping which requires specialized sequences and often a series of breath holds. T1 SIR is typically calculated as a ratio of the measured signal intensity in the pancreas versus measured signal intensity in either the spleen or skeletal muscle on the same image. Literature exists proposing reference and cutoff values for pancreas:spleen signal intensity ratios in adults affected with pancreatitis. Specifically, Tirkes et al. showed mean T1 SIR of 1.03 (pancreas:spleen) and 0.98 (pancreas:muscle) to define chronic pancreatitis in adults [7]. The same authors also showed healthy patients with no history of pancreatitis to have a mean pancreas:spleen SIR of 1.4 [8]. Additionally, these same authors showed a pancreas:spleen SIR of 1.2 to have a sensitivity of 77% and a specificity of 83% (AUC = 0.89) for detecting decreased exocrine function in adults [8].

While there is a growing body of evidence around T1 SIR as a marker of pancreatitis, there is no consensus around specific sampling methods for generation of the T1 SIR. We set out to explore the impact of MRI sequence, sampling location, and region of interest (ROI) shape on quantification T1 SIR in a pediatric and young adult sample.

## Materials and methods

This was a retrospective analysis of T1-weighted fat saturated MR images previously collected for clinical care from a consecutive series of the most recent 200 patients imaged at Cincinnati Children’s Hospital Medical Center between 2018 and 2023. No age restriction was applied. All patients had been imaged on Philips MRI machines. The institutional review board at Cincinnati Children’s Hospital Medical Center provided an exempt determination with a waiver of need to document informed consent for this work. Imaging data were included based on the availability of both a respiratory triggered 3D radial (radial) 2-point Dixon RF spoiled gradient recalled echo (GRE) and 3D 2-point breath hold mDixon RF spoiled GRE sequence in the clinically acquired magnetic resonance cholangiopancreatography examination. There was no clinical exclusion criteria.

Images had been acquired on one of five MRI scanners (four 1.5 T, one 3 T scanner). This included two different models of 1.5 T scanners (Ambition and Ingenia, Philips Healthcare; Best, The Netherlands) and one 3 T scanner (Ingenia, Philips Healthcare; Best, The Netherlands). All images had been acquired with a dedicated torso coil consisting of 28 elements (16 anterior, 12 posterior).

Representative acquisition parameters for the respiratory navigated 3D radial 2 point mDIXON RF spoiled gradient recalled echo sequence included: TR = 7.3 ms, TE (N = 2) = 2.0;4.4 ms, flip angle = 12°; FOV, 320 × 320 mm^2^; matrix, 320 × 320, TFE factor = 38, Radial % = 200, SENSE acceleration factor (in plane phase encode/slice phase encode) = 1/2.2, Half Fourier (slice) = 0.8, slice thickness = 4 mm; acquisition time ≥ 3 min 46 s (navigator gated used for respiratory compensation). Representative acquisition parameters for the breath-hold 3D 2-point mDIXON RF spoiled gradient echo sequence: TR = 5.3 ms, TE (N = 2) = 1.74;3.6 ms, flip angle = 15°; FOV, 320 × 320 mm^2^; matrix, 212 × 212, SENSE acceleration factor (in plane phase encode/slice phase encode) = 2/1.8, slice thickness = 4 mm, Half Fourier (In plane Phase encode/Slice) = 0.675/0.75; acquisition time: ~ 16–17 s. For both sequences, the images were acquired in the axial plane and positioned to cover the liver dome to the iliac crest. The water only images (analogous to a traditional fat satutrated image) were generated for each mDIXON sequence and subsequently used for the signal intensity measurements.

### Image analysis

**Fig. 1:**
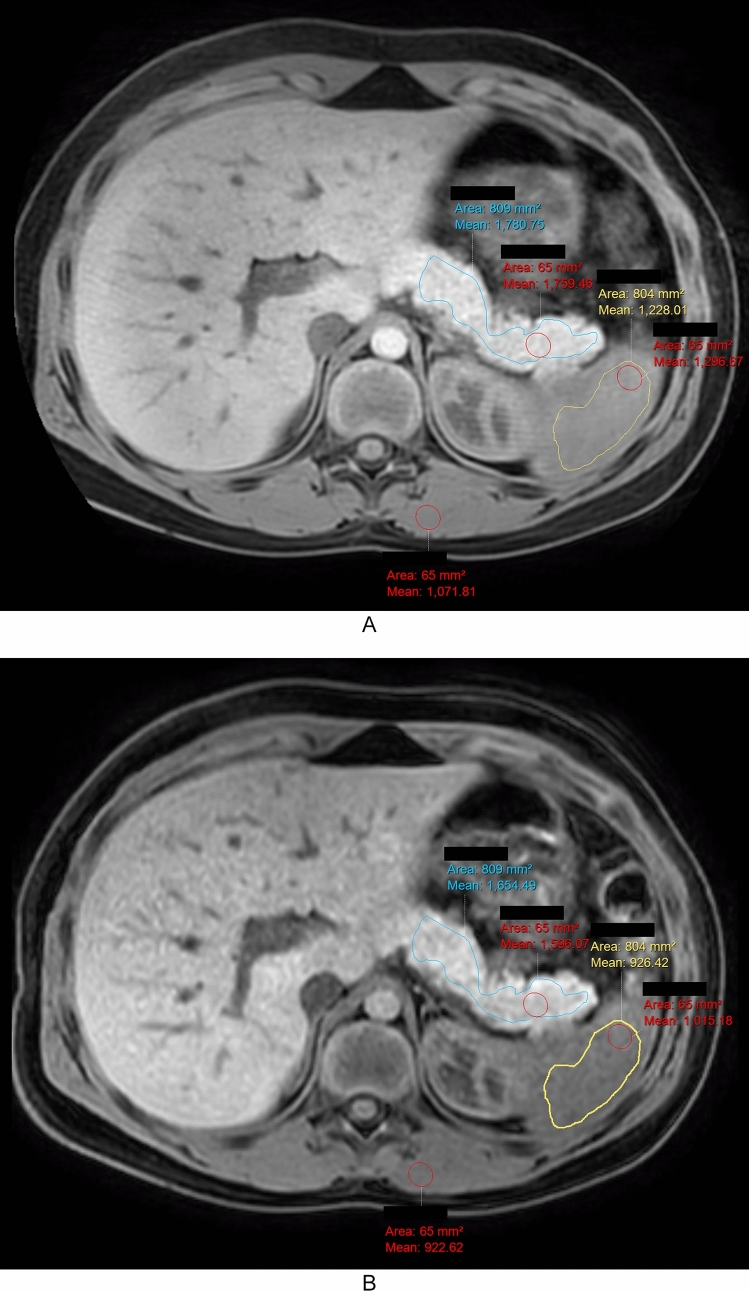
Representative images showing region of interest placement for T1 signal intensity ratio measurement in a 10 year old boy with history of acute recurrent pancreatitis. **A** 3D radial 2-point Dixon gradient recalled echo (GRE) image and **B** 3D breath-hold 2-point Dixon GRE image show similar area irregular pancreas (blue outline) and spleen (yellow outline) regions of interest (ROI) and size-matched ovoid ROIs (red outline) in the pancreas, paraspinal muscle, and spleen. Based on the ovoid ROIs, pancreas:spleen T1 SIR are 1.35 and 1.57, respectively and pancreas:muscle T1 SIR are 1.64 and 1.73, respectively

A single observer (<BLINDED FOR REVIEW>; medical student) measured mean signal intensity in the pancreas, spleen, and left paraspinal muscles on each of the two axial T1-weighted image series using manually placed regions of interest (ROIs) (Intellispace; Phillips Healthcare, Best, The Netherlands) (Fig. 1). ROI measurements were made on anatomically matched slices from each of the T1-weighted MRI sequences.

ROI placement proceeded as follows: On a single image, large, irregular ROIs were placed to encompass (1) as much as possible of the pancreas body and tail, and (2) an approximately similar area (in mm^2^) of the spleen. ROIs were drawn to avoid the margins of either organ, areas of artifact, large vessels, lesions, and dilated pancreas ducts (if present). The same observer, on the same image, also placed ovoid ROIs in the pancreas tail, spleen, and left paraspinal muscle. The observer placed an additional ovoid ROI on a different axial image in the head/uncinate process of the pancreas. The process of drawing ovoid ROIs began by drawing the largest possible ovoid ROI in the tail of the pancras while avoiding the margins of the pancreas, the pancreatic duct if dilated, areas of artifact, large vessels, and lesions. Size-matched ovoid ROIs were then propagated to the other measurement sites. All ROI placements were reviewed by a board-certified pediatric radiologist (A.T.T., 10 years of post-fellowship experience) to ensure correct placement.

### Statistical analysis

Based on the various ROIs placed, multiple different T1 SIR were calculated by dividing mean pancreas signal intensity by mean reference organ signal intensity for the pairs of ROIs (Table 1). Patient demographics were summarized with counts and percentages or means and standard deviation when appropriate. Agreement between T1 SIR measurements was calculated based on intraclass correlations coefficients (ICCs) for absolute agreement and Bland Altman difference analyses. 95% confidence intervals were calculated. ICC was interpreted according to the following scheme: 0–0.49 poor agreement; 0.50–0.74, moderate agreement; 0.75–0.90, good agreement; more than 0.90, excellent agreement. [9] All analyses were performed using MedCalc Statistical Software version 22.014.

**Table 1:** Mean T1 signal intensity ratio (with standard deviation) based on MRI sequence and region of interest shape and placement

	Pancreas tail ovoid:spleen ovoid	Pancreas head/uncinate ovoid:spleen ovoid	Pancreas tail ovoid:muscle ovoid	Pancreas head/uncinate ovoid:muscle ovoid	Pancreas body/tail irregular:spleen irregular	Pancreas body/tail irregular:muscle ovoid
3D radial 2-point Dixon GRE (water image)	1.45 ± 0.20	1.44 ± 0.25	1.53 ± 0.27	1.51 ± 0.26	1.44 ± 0.21	1.53 ± 0.27
3D BH 2-point Dixon	1.54 ± 0.28	1.53 ± 0.31	1.49 ± 0.34	1.47 ± 0.29	1.50 ± 0.26	1.46 ± 0.3

*BH* breath-hold, *GRE* gradient recalled echo

## Results

After exclusion of 71 examinations that included only one of the two T1-weighted sequences of interest, a total of 118 unique patients with 119 MRI examinations were included. Mean participant age was 13.7 ± 5.6 years (range 0.2–28.3 years) and 57 (48.3%) participants were female. The indication for clinical MR was pancreas pathology in 47.4% (n = 56) and liver/biliary tree pathology in 40.0% (n = 46). Indications for the remaining patients were: abdominal pain (n = 8), tumor (n = 5), and other (n = 3). A total of 75 (63.6%) examinations had been acquired on 1.5 T scanners and 43 (36.4%) had been acquired on 3 T scanners. Mean T1 SIR ranged from 1.44 to 1.45 for pancreas:spleen and 1.51 to 1.53 for pancreas:muscle on the radial T1 images and ranged from 1.50 to 1.54 for pancreas:spleen and 1.46 to 1.49 for pancreas:muscle on the breath-held T1 images (Table 1).

### Within sequence T1 SIR agreement based on ROI shape (Table 2***)***

**Table 2:** Within sequence agreement for measured T1 signal intensity ratio based on region of interest (ROI) placement and shape

	Pancreas tail ovoid:spleen ovoid	Pancreas head/uncinate ovoid:spleen ovoid	Pancreas tail ovoid:muscle ovoid	Pancreas head/uncinate ovoid:muscle ovoid	Pancreas body/tail irregular:spleen irregular	Pancreas body/tail irregular:muscle ovoid
Pancreas tail ovoid:spleen ovoid	*3D Radial*	0.67 (0.57 to 0.76) *0.0085 (− 0.35 to 0.37)*	0.41 (0.24 to 0.55) *− 0.084 (− 0.58 to 0.41)*	0.36 (0.19 to 0.50) *− 0.065 (− 0.57 to 0.44)*	0.90 (0.86 to 0.93) *− 0.0074 (− 0.18 to 0.19)*	0.42 (0.25 to 0.56) *− 0.082 (− 0.57 to 0.41)*
	*2pt BH Dixon*	0.61 (0.49 to 0.71) *0.0041 (− 0.51 to 0.52)*	0.61 (0.4 + 9 to 0.71) *0.048 (− 0.48 to 0.58)*	0.39 (0.23 to 0.53) *0.066 (− 0.54 to 0.68)*	0.91 (0.86 to 0.94) *0.036 (− 0.18 to 0.25)*	0.57 (0.43 to 0.68) *0.074 (− 0.45 to 0.59)*
Pancreas head/uncinate ovoid:spleen ovoid		*3D Radial 2pt Dixon*	0.050 (− 0.11 to 0.22) *− 0.093 (− 0.79 to 0.61)*	0.47 (0.31 to 0.60) *− 0.073 (− 0.58 to 0.43)*	0.72 (0.62 to 0.80) *− 0.0011 (− 0.35 to 0.34)*	0.17 (0 to 0.33) *− 0.091 (− 0.74 to 0.56)*
		*3D BH 2pt Dixon*	0.10 (− 0.081 to 0.27) *0.044 (− 0.81 to 0.90)*	0.50 (0.35 to 0.62) *0.062 (− 0.52 to 0.64)*	0.64 (0.52 to 0.73) *0.031 (− 0.45 to 0.51)*	0.16 (− 0.012 to 0.33) *0.070 (− 0.71 to 0.85)*
Pancreas tail ovoid:muscle ovoid			*3D Radial 2pt Dixon*	0.76 (0.77 to 0.82) *0.019 (− 0.33 to 0.38)*	0.46 (0.29 to 0.60) *0.092 (− 0.39 to 0.57)*	0.96 (0.94 to 0.97) *0.0022 (− 0.15 to 0.15)*
			*3D BH 2pt Dixon*	0.70 (0.59 to 0.78) *0.018 (− 0.46 to 0.49)*	0.59 (0.45 to 0.69) *− 0.013 (− 0.55 to 0.53)*	0.95 (0.93 to 0.97) *0.026 (− 0.16 to 0.22)*
Pancreas head/uncinate ovoid:muscle ovoid				*3D Radial 2pt Dixon*	0.52 (0.36 to 0.64) *0.072 (− 0.37 to 0.52)*	0.84 (0.78 to 0.88) *− 0.017 (− 0.31 to 0.27)*
				*3D BH 2pt Dixon*	0.48 (0.33 to 0.61) *− 0.031 (− 0.58 to 0.52)*	0.77 (0.68 to 0.83) *0.0076 (− 0.39 to 0.40)*
Pancreas body/tail irregular:spleen irregular					*3D Radial 2pt Dixon*	0.55 (0.37 to 0.68) *− 0.090 (− 0.52 to 0.35)*
					*3D BH 2pt Dixon*	0.65 (0.53 to 0.74) *0.038 (− 0.43 to 0.50)*

Cells contain results for intraclass correlation coefficients (ICC) with 95% confidence intervals and (in italics) mean differences with 95% limits of agreement based on Bland–Altman difference analysis

3D radial 2pt Dixon = 3D Radial 2-point Dixon; 3D BH 2pt Dixon = 3D breath-hold 2-point Dixon

For the radial sequence, agreement was good for pancreas:spleen T1 SIR sampled using irregular ROIs in both organs versus ovoid ROIs in both organs (ICC = 0.90, mean difference < − 0.01). For the breath-held sequence agreement was excellent for pancreas:spleen T1 SIR sampled using irregular ROIs in both organs versus ovoid ROIs in both organs (ICC = 0.91, mean difference 0.04).

Agreement was excellent on both the radial and breath-held sequences for pancreas: muscle T1 SIR sampled using an irregular versus ovoid pancreatic ROI (Radial: ICC = 0.96, mean difference < 0.01; Breath-held: ICC = 0.95, mean difference 0.03).

### Within sequence T1 SIR agreement based on ROI location in the pancreas (Table 2)

**Fig. 2:**
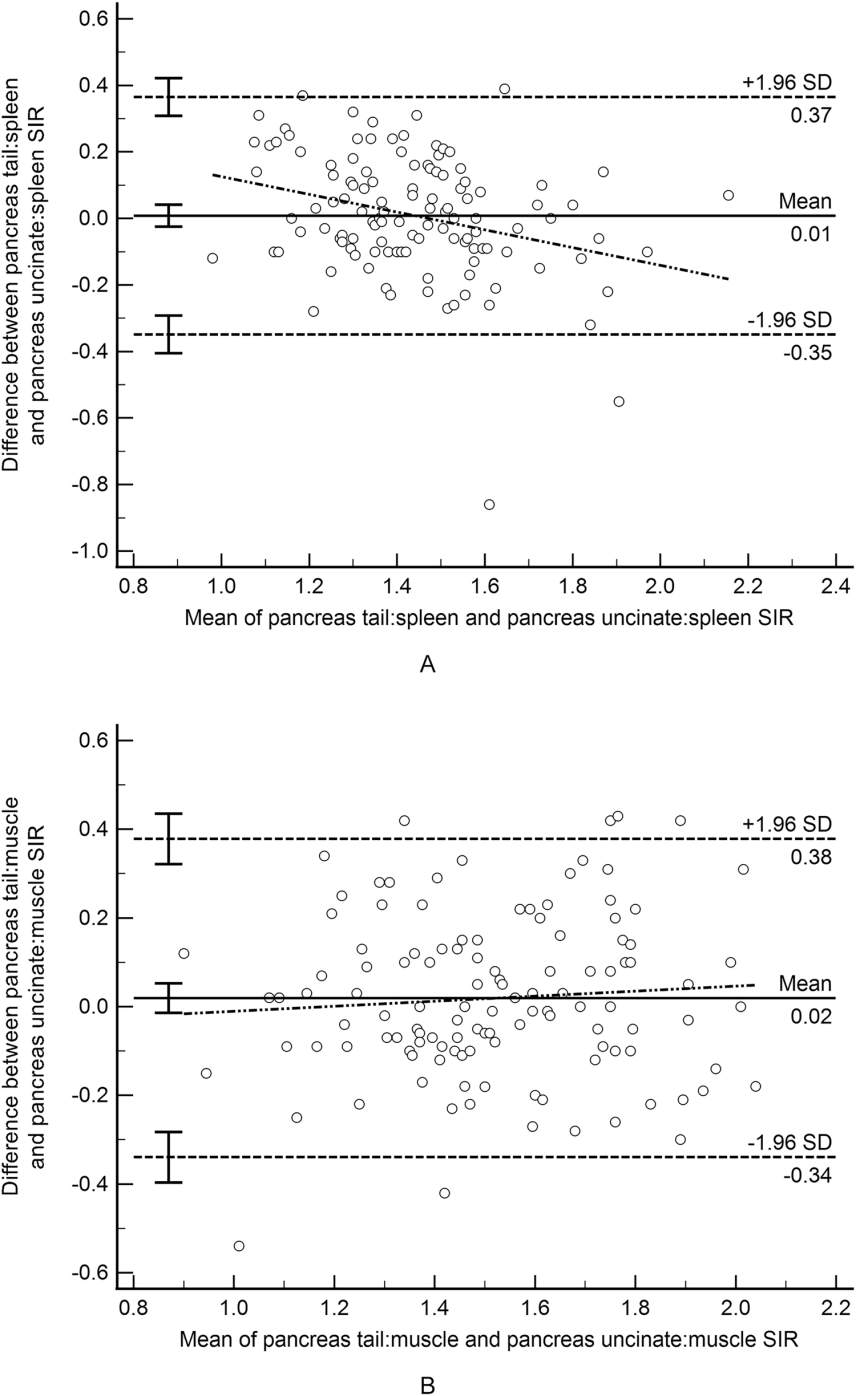
Bland–Altman difference plot demonstrating T1 signal intensity ratio (SIR) agreement based on ovoid region of interest placement in the tail versus head/uncinate process of the pancreas on the 3D radial 2-point Dixon gradient recalled echo images. **A** Pancreas:spleen SIR; **B** Pancreas:muscle T1 SIR

One patient had a prior Whipple procedure precluding measurement of signal intensity in the head/uncinate. For the remaining 117 patients, agreement for T1 SIR based on sampling the pancreas tail versus head/uncinate using ovoid ROIs was moderate for both the radial sequence (pancreas:spleen: ICC = 0.67, mean difference < 0.01, and pancreas:muscle: ICC = 0.76, mean difference 0.19; Fig. 2) and for the breath-held sequence (pancreas:spleen: ICC = 0.61, mean difference < 0.01, and pancreas:muscle: ICC = 0.70, mean difference 0.018).

### Within sequence T1 SIR agreement based on reference organ (Table 2)

**Fig. 3:**
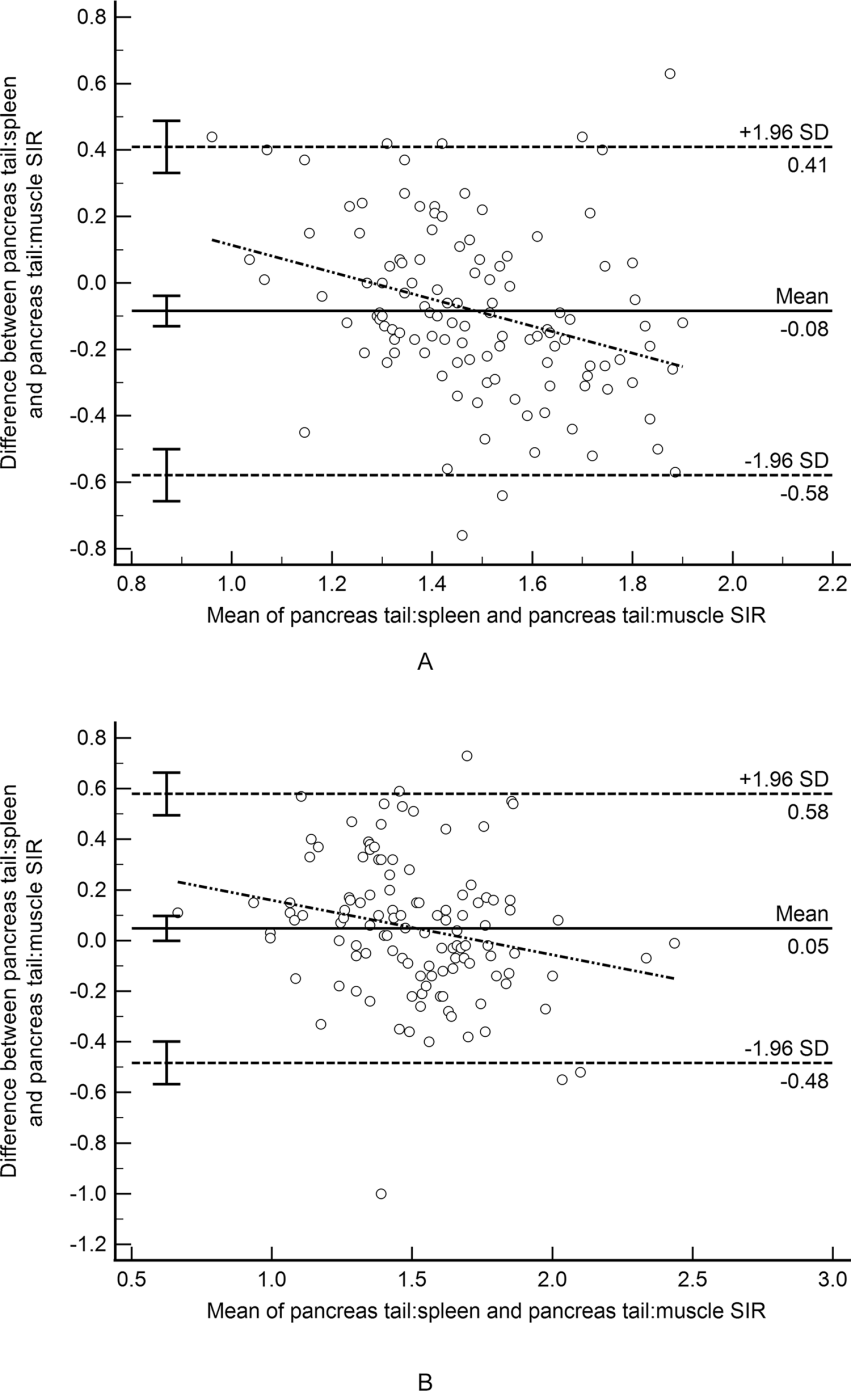
Bland–Altman difference plot demonstrating T1 signal intensity ratio (SIR) agreement based on an ovoid region of interest (ROI) in the pancreas tail and an ovoid ROI in either the spleen or paraspinal muscle as the reference organ. **A** T1 SIR from 3D radial 2-point Dixon gradient recalled echo (GRE) images; **B** T1 SIR from 3D breath-hold 2-point Dixon GRE images

T1 SIR agreement based on reference to the spleen versus muscle was poor for the radial sequence when sampling the tail (ICC = 0.41, mean difference − 0.08; Fig. 3) and head/uncinate process (ICC = 0.47, mean difference − 0.07) and moderate for the breath-held sequence when sampling the tail (ICC = 0.61, mean difference 0.05; Fig. 3) and head/uncinate process (ICC = 0.50, mean difference 0.06).

### T1 SIR agreement based on MRI sequence

**Fig. 4:**
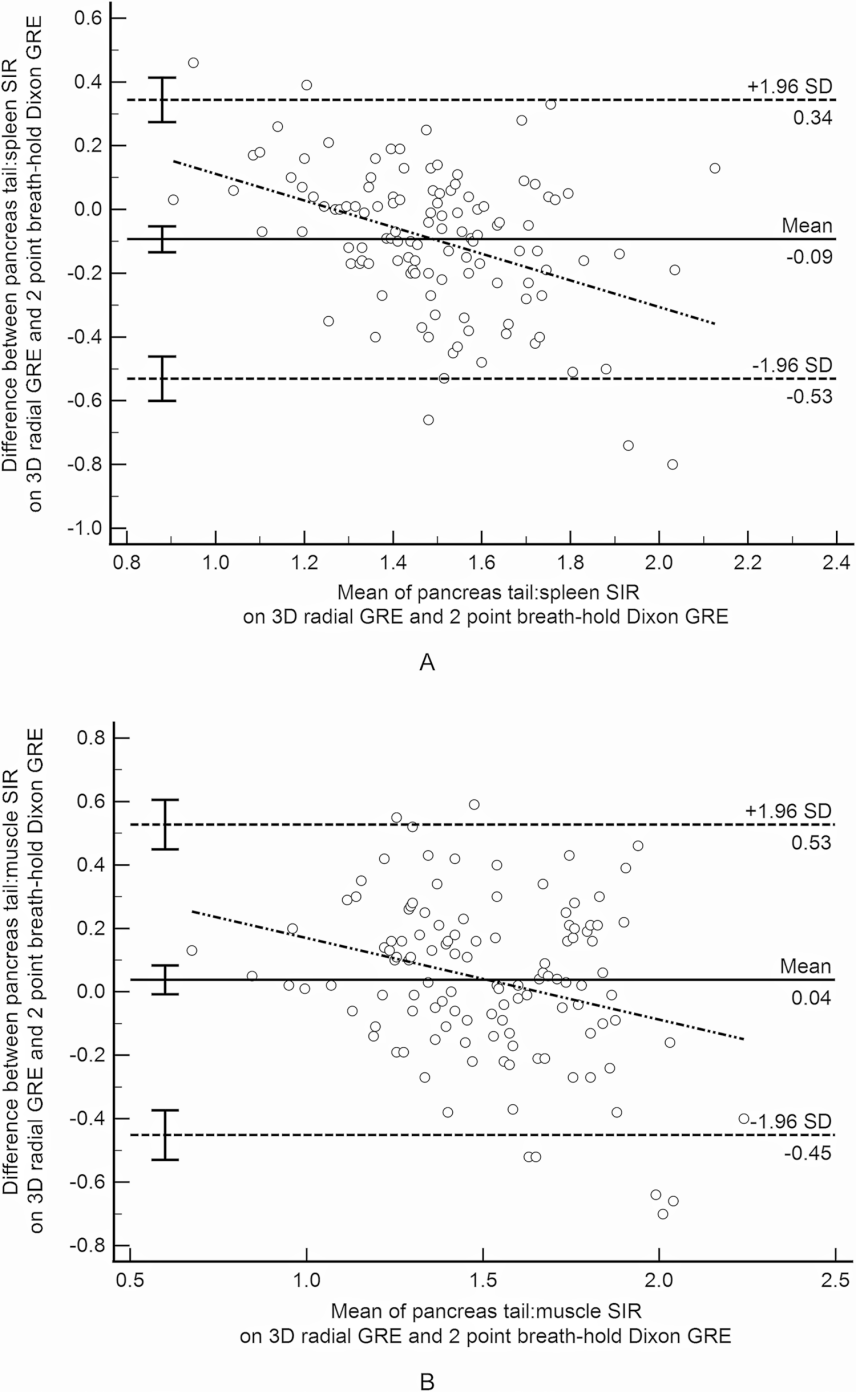
Bland–Altman difference plot demonstrating T1 signal intensity ratio agreement between the 3D radial 2-point Dixon gradient recalled echo (GRE) and 3D breath-hold 2-point Dixon GRE sequences. **A** SIR for ovoid regions of interest in the pancreas tail and spleen; **B** SIR for ovoid regions of interest in the pancreas tail and paraspinal muscle

T1 SIR agreement was moderate between the radial and breath-held sequences for all ROI shape, pancreas location, and reference organ combinations with ICCs between 0.55 and 0.72 and mean differences between 0.04 and 0.09 (Table 3; Fig. 4).

**Table 3:** Between sequence agreement for measured T1 signal intensity ratio by region of interest (ROI) location and shape

ROI	3D radial vs.3D BH 2pt Dixon
Pancreas tail ovoid:spleen ovoid	0.55 (0.37 to 0.68) *− 0.093 (− 0.53 to 0.34)*
Pancreas head/uncinate ovoid:spleen ovoid	0.64 (0.49 to 0.75) *− 0.086 (− 0.52 to 0.34)*
Pancreas tail ovoid:muscle ovoid	0.66 (0.54 to 0.75) *0.038 (− 0.45 to 0.53)*
Pancreas head/uncinate ovoid:muscle ovoid	0.66 (0.55 to 0.76) *0.045 (− 0.38 to 0.47)*
Pancreas body/tail irregular:spleen irregular	0.70 (0.56 to 0.79) *− 0.089 (− 0.50 to 0.33)*
Pancreas body/tail irregular:muscle ovoid	0.72 (0.60 to 0.80) *0.062 (− 0.35 to 0.47)*

Results are presented as intraclass correlation coefficients with 95% confidence intervals and mean difference with 95% limits based on Bland–Altman analysis (italics)

3D radial 2pt Dixon = 3D radial 2-point Dixon; 3D BH 2pt Dixon = 3D breath-hold 2-point Dixon

## Discussion

T1 signal loss is a recognized MRI finding of chronic pancreatitis and native T1 signal intensity, and calculated T1 SIR have received attention as a potential biomarkers for chronic pancreatitis [5]. Tirkes et. al. reported that pancreas:spleen T1 SIR was associated with disease severity in chronic pancreatitis [7]. Fukada et al. also found mean pancreas:muscle T1 SIR to decrease with the progression of chronic pancreatitis and to predict pancreatic fistula and exocrine insufficiency [10]. Based on these findings and other literature, T1 SIR has been included in reporting standards for adult chronic pancreatitis [7]. While there is a growing body of evidence supporting T1 SIR as a marker of pancreatitis, there is no consensus around specific sampling methods for generation of the T1 SIR, and technical factors may impact measured T1 SIR. Technical variability in T1 SIR is potentially relevant both in terms of initial diagnosis of pancreatitis using T1 SIR thresholds and in terms of longitudinal tracking of T1 SIR as a potential marker of disease progression.

Our results show good to excellent agreement in measured T1 SIR based on small ovoid vs. larger irregular ROIs drawn in the pancreas suggesting that the shape and size of the pancreas ROI do not meaningfully impact T1 SIR and that standardization of this is not needed. However, agreement was only moderate for measured T1 SIR based on the location of ROI placement (head/uncinate versus body/tail) within the pancreas and agreement was poor to moderate based on use of the spleen versus muscle as the reference organ. Variation in measured T1 SIR based on location of the ROI in the pancreas may reflect non-uniform involvement by disease as well as slice-to-slice variation in MRI signal. It is therefore important that ROI placement within the pancreas is standardized at initial assessment and replicated throughout follow up of a given patient. Further, it is important that the the reference organ used be standardized to reduce technique-related variation in measured T1 SIR. Optimally, ROI placement and reference organ standardization would be based on evidence-based clinical and research practice, to allow application of evidence-based threshold T1 SIR values for diagnosis and potentially disease staging. In the absence of evidence-based practice, standardization within institutions/practices should reduce variability in T1 SIR.

Our results also show that agreement is only moderate for T1 SIR between different T1-weighted sequences. Sequence differences reflect differences in acquisition parameters that both influence overall T1 weighted signal intensity in the image as well as influence relative signal intensity between the pancreas and reference organs (spleen and muscle) due to tissue composition differences, thereby impacting measured T1 SIR. Based on the 95% limits of the difference in calculated T1 SIR seen our our study, sequence differences could result in variations in T1 SIR of as much as 0.5 to 0.6. This magnitude of difference is sufficient to impact diagnostic performance of T1 SIR as a marker of pancreatic disease. There is therefore need to standardized the specific T1-weighted sequence used to measure T1 SIR. In the current study, we compared T1 SIR derived from two different T1-weighted, fat-saturated MRI sequences: a respiratory navigated 3D radial 2 point mDIXON RF spoiled gradient recalled echo sequence and breath-hold 3D 2-point mDIXON RF spoiled gradient echo sequence. In clinical practice, the breath-hold GRE sequence is likely the most frequently used and this is the sequence recommended for T1 SIR measurements by the Consortium for the Study of Chronic Pancreatitis, Diabetes, and Pancreatic Cancer (CPDPC) in their reporting standards for chronic pancreatitis. The required breath old for this sequence is relatively long (on the order of 10–15 s) and may not be achievable in a reliable manner in pediatric patients or in acutely ill patients. Thus, motion artifact will likely impact measured signal intensity and the associated calculated T1 SIR. The respiratory navigated 3D radial sequence allows imaging without a breath-hold but requires a longer scan duration and, depending on implementation, could introduce radial artifact and image blur. Both of these sequences are potentially viable approaches to obtaining T1-weighted images to allow measurement of a T1 SIR and other sequences, including non-fat saturated T1-weighted sequences are available. Ideally there would be evidence-driven consensus in the field on which T1-weighted, fat-saturated sequence to use for measurement of T1 SIR. Until this is achieved, sequence standardization at the institutional level is important to minimize technical variation.

In the absence of standardized methods for collecting T1 SIR, and given the variability in measured T1 SIR in our study, multicenter studies, definition of normative values, and definition of threshold values for disease will be difficult to achieve. These problems are exemplified by the fact that prior literature suggests that T1 SIR thresholds defined for adults may not be applicable to pediatrics. Specifically, McCleary et al. showed that pancreas:spleen T1 SIR cutoffs previously described by Tirkes et al. to suggest exocrine efficiency and pancreatic disease in adult populations are likely too restrictive for pediatric populations [4, 7]. Specifically, McCleary et al. found pancreas:spleen T1 SIR in healthy children to be commonly below the threshold Tirkes et al. defined for pancreatic insufficiency. Further, McCleary et al. showed a significant negative correlation between T1 SIR age and height, suggesting the potential need for age specific cutoff measures for T1 SIR [4]. It is unknown if these observed discrepancies reflect differences between children and adults or differences between the sequences used to calculate T1 SIR, or both.

While our study is insufficient to define optimal sampling technique and to define the clinical impact of observed variation in measured T1 SIR, it does identify the need for standardization. Based on this, and until consensus on optimal technique can be reached, we suggest the following:

1. Institutions/practices should choose and uniformly use a single T1-weighted, fat-saturated sequence and fixed set of acquisition parameters for measurement of T1 SIR. Choosing the 3D 2 point mDIXON RF spoiled gradient recalled echo sequence, recommended by the CPDPC, either with respiratory navigated radial acquisition or breath holding is suggested.
2. Institutions/practices should choose, and uniformly use a single reference organ for calculation of T1 SIR. Both spleen and muscle have advantages and disadvantages as reference organs. Spleen is recommended as the reference organ, as the greatest body of literature concerning T1 SIR uses spleen as a reference organ.
3. Size-matched ovoid regions of interest (chosen over irregular ROIs for simplicity) should be used to measure T1 SIR in the pancreas and reference organ on the same image.
4. In patients with diffuse disease, a single pancreas ROI may be adequate for measurement of T1 SIR. In patients with non-uniform disease (e.g. segmental/focal disease), or when diffuse versus focal disease cannot be reliably distinguished, multiple pancreas ROIs with same-image matched reference organ ROIs should be used with the lowest T1 SIR reported.
5. In follow-up, pancreas ROIs used to calculate T1 SIR should be matched in size and location to ROIs used in prior examinations of the same patient.

Adoption of these recommendations should create institutional standardization of T1 SIR sampling, reducing technical variation in calculated T1 SIR and facilitating longitudinal assessment of patient-level change in T1 SIR.

Our study is limited by the fact it is a retrospective study using clinically acquired T1-weighted images. Measured variation may have been less if the acquired images had been carefully standardized (see recommendations above). However, the sample included is representative of real-world clinical practice. The retrospective design of our study is also limited in that we could not compare T1 SIR between fat saturated and non-fat saturated T1-weighted sequences and we could not systematically compare T1 SIR between MRI field strengths. While fat saturated T1-weighted sequences are considered part of a minimum standard MRI protocol for imaging pancreatitis, not all sites will choose to employ fat saturation [11]. Our study is additionally limited by the fact it includes a convenience sample with a predominance of patients without pancreatic disease. Heterogenous disease and disease distribution in the pancreas could increase the measured variability in T1 SIR, particularly for comparison of T1 SIR based on ROI placement within the pancreas. Finally, only a single observer made measurements for calculation of T1 SIR precluding us from assessing the impact of interobserver variation on measured T1 SIR in the current study. Prior work has shown interobserver agreement on T1 SIR to be moderate for pancreas:spleen and good for pancreas:muscle [3].

In conclusion, the size and shape of the ROI used to sample the pancreas on T1-weighted MR images to generate T1 SIR in children and young adults likely does not meaningfully change the measured T1 SIR but the location sampled (pancreatic head/uncinate vs. body/tail), the reference organ used (spleen vs. muscle) and the specific MRI sequence chosen likely do meaningfully change the measured T1 SIR, potentially impacting disease diagnosis and staging. Our results suggest that consensus on T1 SIR sampling technique is needed and that, in the absence of this, individual institutions/practices should standardize their sampling technique. We have provided recommendations for individual practices to achieve this.

## Data Availability

Manuscript data are available from the authors upon reasonable request.
